# Trends in the pre-operative diagnosis and surgical management of axillary lymph node metastases in women with screen-detected breast cancer

**DOI:** 10.1016/j.breast.2023.103593

**Published:** 2023-10-21

**Authors:** Lucien E.M. Duijm, Luc J.A. Strobbe, Vivian van Breest Smallenburg, Clemence L. op de Coul-Froger, Wikke Setz-Pels, Willem Vreuls, Hermen C. van Beek, Rob M.G. van Bommel, Adri C. Voogd

**Affiliations:** a Department of Radiology, Canisius Wilhelmina Hospital, Weg door Jonkerbos 100, 6532, SZ, Nijmegen, the Netherlands; b Department of Surgical Oncology, Canisius Wilhelmina Hospital, Weg door Jonkerbos 100, 6532, SZ, Nijmegen, the Netherlands; c Department of Radiology, Jeroen Bosch Hospital, Henri Dunantstraat 1, 5223, GZ, ‘s-Hertogenbosch, the Netherlands; d Department of Radiology, Bernhoven Hospital, Nistelrodeseweg 10, 5406, PT, Uden, the Netherlands; e Department of Radiology, Catharina Hospital, Michelangelolaan 2, 5623, EJ, Eindhoven, the Netherlands; f Department of Pathology, Canisius Wilhelmina Hospital, Weg door Jonkerbos 100, 6532, SZ, Nijmegen, the Netherlands; g Department of Radiology, Maxima Medical Center, De Run 4600, 5504, DB Veldhoven, the Netherlands; h Department of Radiology, St Anna Hospital, Bogardeind 2, 5664, EH, Geldrop, the Netherlands; i Department of Epidemiology, Maastricht University, PO Box 616, 6200, MD, Maastricht, the Netherlands

**Keywords:** Axilla, Biopsy Fine-needle, Biopsy Large-core needle, Breast neoplasms, Lymph node excision, Sensitivity and specificity, Sentinel lymph node biopsy

## Abstract

**Aim:**

The aim of the current study was to investigate time-trends in pre-operative diagnosis and surgical treatment of axillary lymph node metastases in breast cancers detected at screening mammography.

**Methods:**

We included all women who underwent screening mammography in the South of the Netherlands between 2005 and 2020. During a follow-up period of at least two years, data on clinical radiological examinations, biopsy procedures and surgical interventions were obtained. The 15 years of inclusion were divided into five cohorts of three years each.

**Results:**

Of the 4049 women with invasive breast cancer, 22.1 % (896/4049) had axillary lymph node metastasis at pathology (ALN+). Percutaneous axillary biopsy was performed in 39.6 % (355/896) of these women, with the proportions of fine needle aspiration biopsy (FNAB) decreasing from 97.6 % (40/41) in 2005–2007 to 41.6 % (37/89) in 2017–2019 and core needle biopsy (CNB) rising from 2.4 % (1/41) in 2005–2007 to 58.4 % (52/89) in 2017–2019 (P < 0.001). Sensitivity of FNAB and CNB was comparable (77.4 % (188/243, 95%CI = 71%–82 %) versus 82.4 % (103/125), 95%CI = 74%–88 %) (P = 0.26). Pre-operative confirmation of ALN + by percutaneous biopsy ranged from 27.3 % (56/205) in 2011–2013 to 39.0 % (80/205) in 2017–2019, with no significant trend changes over time (P = 0.103). The proportion of ALN + women who underwent axillary lymph node dissection (ALND) decreased from 96.0 % (97/101) in 2005–2007 to 16.6 % (34/205) in 2017–2019 (P < 0.001).

**Conclusion:**

Pre-operative confirmation of axillary lymph node metastasis by ultrasound-guided biopsy did not rise despite the increased use of CNB at the expense of less invasive FNAB. A significant reduction in ALND was observed through the years.

## Highlights

• Fine-needle aspiration biopsy was increasingly replaced by core-needle biopsy for sampling of suspicious axillary lymph nodes.
• The sensitivity of fine-needle aspiration biopsy and core-needle biopsy was comparable.
• The yield of percutaneous axillary biopsy in case of screen-detected breast cancer did not improve over the years.
• The proportion of lymph node positive women that underwent axillary lymph node dissection significantly decreased over time.

## 1 Introduction

Screening mammography aims to detect breast cancer at an early stage. The combination of this early detection with improved therapy has resulted in a decreased breast cancer mortality [[1], [2], [3]]. The Netherlands has a long history of biennial screening mammography, with a nation-wide programme fully implemented in 1998 [4,5]. Although a majority of cancers detected by screening are small and low grade, up to 20 % of screen-detected cancers prove to have metastasized to the axillary region [6]. In most European countries, pre-operative confirmation of axillary lymph node metastases is obtained by percutaneous biopsy of suspicious lymph nodes seen at axillary ultrasonography (AUS). Biopsy may be performed using fined needle aspiration biopsy (FNAB) or core needle biopsy (CNB). Single centre studies report conflicting results as to whether CNB has a higher sensitivity than FNAB for axillary staging [7,8]. Sentinel node biopsy can be omitted if axillary lymph node metastasis has been confirmed by percutaneous biopsy [9]. In the past, women with axillary metastasized breast cancer routinely underwent axillary lymph node dissection (ALND). Unfortunately, ALND may be complicated by a myriad of short- and long-term adverse events of which lymphedema is the most feared one [10]. Several studies have reported that ALND can be omitted and replaced by regional nodal irradiation following sentinel lymph node biopsy in breast cancers with limited axillary metastasized disease [11,12]. A population-based study in the Netherlands found a decreased use of ALND in a mix of both asymptomatic and symptomatic women with a low metastatic burden [13]. Screening mammography programmes aim to include only asymptomatic women, whereas women with breast complaints should be primarily assessed at dedicated breast units. Screen-detected breast cancers are usually much smaller than breast cancers detected in symptomatic women. Moreover, these tumours tend to be more often estrogen receptor positive and lower graded. The detection and subsequent treatment of small and less aggressive tumours may lead to overdiagnosis and overtreatment, which are well known disadvantages of screening mammography [14,15]. It is therefore important, especially in this breast cancer population, to minimize the overtreatment by ALND. The purpose of the current population-based study was to describe time trends in the use of FNAB and CNB for axillary staging in women with screen-detected breast cancer, as well as comparing the yield of these biopsy techniques. Furthermore, we determined time trends in the application of ALND in screen-detected breast cancers metastasized to the axilla.

## 2 Patients and methods

### 2.1 Study population and design of the screening mammography programme

The Dutch screening programme offers free biennial screening mammography to women aged 50–75 years. We included a consecutive series of women who were screened in a Southern screening region of the Netherlands between January 1, 2005 and January 1, 2020. Prior to attendance, women gave informed consent that their data could be used for quality assurance of the screening programme and for scientific purposes. Women could refrain from this consent using an opt-out construction. Only three recalled women did so and they were excluded from analysis. Ethical approval was not required for the current study, according to the Dutch Central Committee on Research involving Human Subjects (CCMO).

Details of our screening programme have been reported previously [4,16]. In summary, the screening mammograms were obtained at four specialized screening units (three mobile units and one fixed unit) by specialized radiologic technicians. Screen-film mammography was gradually replaced by digital screening mammography in 2009/2010. All screening examinations were double read by certified screening radiologists. Non-blinded double reading (the second reader is not blinded to the first reader's opinion) was replaced by blinded double reading in 2015. A woman was referred to a hospital for further analysis if suspicious findings were detected at screening mammography.

### 2.2 Analysis and follow-up of recalled women

After recall, additional breast imaging was performed at the hospital where the woman was referred to and the findings were classified according to the Breast Imaging Reporting and Data System (BI-RADS) [17,18]. BI-RADS 4 (suspicious) and BI-RADS 5 (malignant) lesions were routinely biopsied. BI-RADS 3 lesions (probably benign) could undergo either biopsy or radiological follow-up, dependent on shared decision between the clinician and the patient. Axillary ultrasound was routinely performed in BI-RADS 4 and 5 findings and in the majority of BI-RADS 3 abnormalities (especially if biopsied). Axillary lymph nodes were biopsied when they showed a cortical thickness exceeding 2.5 mm or atypical morphological characteristics. The type of percutaneous biopsy, either FNAB (21 gauge) or CNB (14, 16 or 18 gauge), and the number of biopsies obtained at CNB, was at the discretion of the radiologist who performed the biopsy procedure. The screening secretariat, one of the authors (LD) and several radiology residents collected follow-up data at the hospitals that analyzed the recalled women. The majority of these recalls was analyzed in 6 hospitals (97.8 %, 19,950/20,399 recalls). During a follow-up period of two years (until the next biennial screen) all reports on clinical breast imaging, biopsy reports and surgical interventions were obtained. Screen-detected cancers were divided into ductal carcinoma in-situ (DCIS) and invasive cancer. Lobular carcinoma in-situ was not considered malignant. Axillary lymph node metastases at sentinel node biopsy (SNB) and ALND were divided into submicro-metastases N0(i+) (<0.2 mm or isolated tumour cells), micro-metastases N1mi (0.2–2 mm) and macro-metastases N1 (>2 mm) [19]. Lymph nodes were considered negative if they only harbored submicro-metastasis. For the purpose of the current study, we included the tumour with the highest axillary nodal status in case of bilateral breast cancer.

The COVID-19 pandemic urged the screening programme to stop in the beginning of 2020 and screening was restarted in the autumn of the same year. This discontinuity temporarily resulted in extension of the screening interval from two years to almost three years. Therefore, follow-up was likewise extended to three years for women who had been screened in 2018 and 2019.

### 2.3 Statistical analysis

Trends in patients and tumour characteristics, pre-operative confirmation of ALN+, use of FNAB and CNB and application of ALND were studied by 3-year periods: 2005–2007, 2008–2010, 2011–2013, 2014–2016 and 2017–2019. Changes over time and between periods were tested by using chi-square tests. A P-value <0.05 was considered as statistically significant.

## 3 Results

### 3.1 Overall screening outcome

**Fig. 1:**
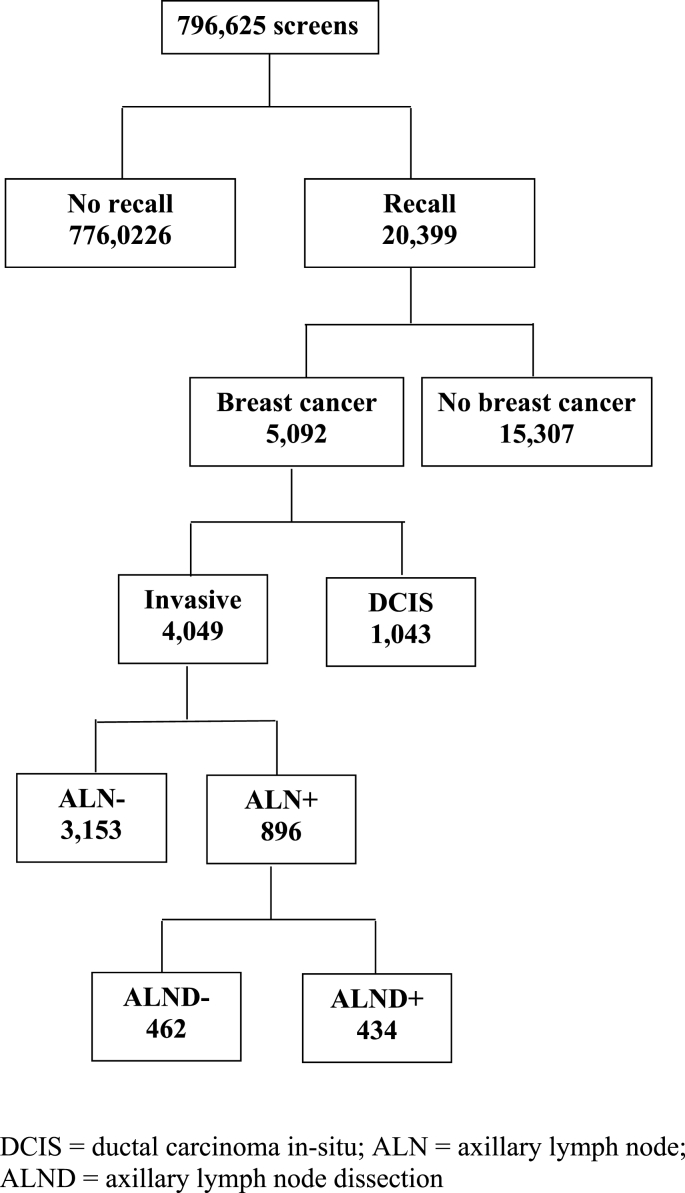
Study population.

A total of 796,625 screens were obtained (84,873 initial screens and 711,752 subsequent screens), resulting in 20,399 recalls (recall rate, 2.6 %) and 5092 women with a screen-detected cancer (6,4 cancers per 1000 screens, Figure). DCIS comprised 20.5 % (1043/5092) of all screen-detected cancers (Table 1 and Fig. 1). Histologically proven axillary metastases were present in 22.1 % (896/4049) of women with invasive breast cancer, ranging from 20.7 % (205/900) in 2017–2019 to 24.3 % (141/580) in 2008–2010 (P = 0.39). Of the ALN + cancers, respectively 12.9 % (116/896), 44.2 % (396/896) and 42.5 % (381/896) were sized ≤10 mm (T1a-b), 11–20 mm (T1c) or >20 mm (T2+). The tumour size was unknown in the remaining 3 women (0.3 %). Most ALN + cancers were Bloom & Richardson grade I or II, estrogen and progesterone receptor positive and Her2/Neu negative. The size distribution of N+ cancers was the only tumour characteristic showing statistically significant differences among the cohorts (P = 0.003, Table 1).

**Table 1:** Overall screening results and tumour characteristics of ALN + screen-detected breast cancers.

Screening year	2005–2007	2008–2010	2011–2013	2014–2016	2017–2019	P-value
Screens, No	100219	123739	178963	197556	196148	
Recall, No (%)	1376 (1.4)	2860 (2.3)	5907 (3.3)	5876 (3.0)	4380 (2.2)	
Cancer detection rate^≠^	5.2	5.9	7.0	6.7	6.4	
Type of screen-detected cancer, No (%)						0.106
DCIS	92 (17.7)	145 (20.0)	281 (22.4)	255 (19.1)	270 (21.4)	
Invasive cancer	428 (82.3)	580 (80.0)	973 (77.6)	1078 (80.9)	990 (78.6)	
Invasive cancers, No	428	580	973	1078	990	
N+ invasive cancers, No (%)	101 (23.6)	141 (24.3)	205 (21.1)	244 (22.6)	205 (20.7)	0.394
Size of N+ cancers, No (%)						0.003
T1a-b	12 (11.9)	8 (5.7)	39 (19.0)	36 (14.8)	21 (10.2)	
T1c	37 (36.6)	75 (53.2)	87 (42.4)	96 (39.3)	101 (49.3)	
T2+	51 (50.5)	58 (41.1)	79 (38.5)	112 (45.9)	81 (39.5)	
Unknown*	1 (1.0)	0	0	0	2(1.0)	
B&R of N+ cancers, No (%)						0.313
I	34 (33.7)	51 (36.2)	74 (36.1)	78 (32.0)	72 (35.1)	
II	42 (41.6)	68 (48.2)	92 (44.9)	127 (52.0)	111 (54.1)	
III	18 (17.8)	19 (13.5)	36 (17.6)	37 (15.2)	20 (9.8)	
Unknown*	7 (6.9)	3 (2.1)	3 (1.5)	2 (0.8)	2 (1.0)	
Estrogen receptor status of N+ cancers, No (%)						0.538
ER+	84 (83.2)	123 (87.2)	182 (88.8)	215 (88.1)	185 (90.2)	
ER-	16 (15.8)	17 (12.1)	22 (10.7)	28 (11.5)	19 (9.3)	
Unknown*	1 (1.0)	1 (0.7)	1 (0.5)	1 (0.4)	1 (0.5)	
Progesterone receptor status of N+ cancers, No (%)						0.947
PR+	70 (69.3)	97 (68.8)	150 (73.2)	171 (70.1)	144 (70.2)	
PR-	30 (29.7)	41 (29.1)	54 (26.3)	71 (29.1)	60 (29.3)	
Unknown*	1 (1.0)	3 (2.1)	1 (0.5)	2 (0.8)	1 (0.5)	
Her2/Neu receptor of N+ cancers, No (%)						0.494
Her2/Neu+	14 (13.9)	22 (15.6)	26 (12.7)	31 (12.7)	19 (9.3)	
Her2/Neu-	87 (86.1)	118 (83.7)	178 (86.8)	212 (86.9)	185 (90.2)	
Unknown*	0	1 (0.7)	1 (0.5)	1 (0.4)	1 (0.5)	
Receptor triple negative status of N+ cancers, No (%)						0.661
Yes	10 (9.9)	7 (5.0)	13 (6.3)	16 (6.6)	15 (7.3)	
No	91 (90.1)	133 (94.3)	191 (93.2)	227 (93.0)	189 (92.2)	
Unknown*	0	1 (0.7)	1 (0.5)	1 (0.4)	1 (0.5)	

ALN = axillary lymph node; DCIS = ductal carcinoma in-situ; B&R = Bloom & Richardson. ^≠^Per 1000 screens; *Not included at statistical analysis.

### 3.2 Pre-operative confirmation of axillary lymph node metastasis by percutaneous biopsy

Percutaneous axillary biopsy was performed in 39.6 % (355/896) of women who proved to have axillary metastasized disease, ranging from 35.6 % (73/205) in 2011–2013 to 45.9 % (94/205) in 2017–2019 (P < 0.001, Table 2). There were no statistically significant trends over time (P = 0.243). The proportion of FNAB among all axillary biopsies decreased from 97.6 % (40/41) in 2005–2007 to 41.6 % (37/89) in 2017–2019 while the proportion of CNB rose from 2.4 % (1/41) in 2005–2007 to 58.4 % (52/89) in 2017–2019 (P < 0.001). No statistically significant trend could be observed in the proportion of women with pre-operatively confirmed nodal metastasis (P = 0.103). In pairwise comparison between the periods 2011–2013 and 2017–2019, a higher proportion was observed in the last period: 27.3 % (56/205) in 2011–2013 compared to 39.0 % (80/205) in 2016–2019 (P = 0.012). The overall sensitivity of FNAB and CNB were comparable (77.4 % (188/243; 95 % CI = 71%–82 %) versus 82.4 % (103/125); 95 % CI = 74%–88 %) (P = 0.26). The overall sensitivity of axillary ultrasound for ALN + detection was 32.5 % (291/896).

**Table 2:** Axillary biopsy and axillary lymph node dissection of screen-detected breast cancers.

Screening year	2005–2007	2008–2010	2011–2013	2014–2016	2017–2019	P-value
N+ invasive cancers, No (%)	101 (23.6)	141 (24.3)	205 (21.1)	244 (22.6)	205 (20.7)	0.394
Pre-operative confirmation of axillary metastasis, No (%)	34 (33.7)	40 (28.4)	56 (27.3)	81 (33.2)	80 (39.0)	0.130
Percutaneous axillary biopsy in N+ cancers, No (%)	41 (40.6)	51 (36.2)	73 (36.6)	96 (39.3)	94 (45.9)	0.243
Type of percutaneous axillary biopsy in N+ cancers, No (%)						<0.001^≠^
FNAB	40 (97.6)	45 (88.2)	49 (67.1)	59 (61.5)	37 (39.4)	
Core needle biopsy	1 (2.4)	4 (7.8)	21 (28.8)	34 (35.4)	52 (55.3)	
FNAB + core needle biopsy	0	2 (3.9)	3 (4.1)	3 (3.1)	5 (5.3)	
Sensitivity^a^ of percutaneous axillary biopsy, %						
FNAB	82.5 (33/40)	72.3 (34/47)	73.1 (38/52)	82.3 (51/62)	76.2 (32/42)	0.020
Core needle biopsy	100 (1/1)	100 (6/6)	75.0 (18/24)	81.1 (30/37)	84.2 (48/57)	0.848^≠^
N+ cancers with axillary lymph node dissection, No (%)	97 (96.0)	127 (90.1)	118 (57.6)	58 (23.8)	34 (16.6)	<0.001
Type of axillary lymph node metastasis at surgical specimen, No (%)						0.038
Micro (0.2–2 mm)	21 (20.8)	32 (22.7)	70 (34.1)	71 (29.1)	49 (23.9)	
Macro (>2 mm)	80 (79.2)	109 (77.3)	135 (65.9)	173 (70.9)	156 (76.1)	

^≠^Fisher's Exact Test. FNAB = fine needle aspiration biopsy.

a True positive/true positive + false negative.

### 3.3 ALND in women with lymph node metastasis at percutaneous biopsy or SNB

The proportion of axillary metastasized women who underwent axillary lymph node dissection (ALND) gradually decreased from 96.0 % (97/101) in 2005–2007 to 16.6 % (34/205) in 2017–2019 (P < 0.001). A majority of the ALN + women, 72.9 % (653/896), showed macro-metastases at SN and/or ALND, ranging from 65.9 % (135/205) in 2011–2013 to 79.2 % (80/101) in 2005–2007 (P = 0.038).

## 4 Discussion

In this multi-institutional study we found no increase in the pre-operative confirmation rate of axillary lymph node metastasis over the years, despite the increased use of CNB at the expense of FNAB. The sensitivity of FNAB and CNB were comparable. A significant reduction in the use of ALND was observed in women with ALN + screen-detected cancer.

Several studies have been published on the value of percutaneous biopsy for axillary staging. In a multicentre study of pT1 tumours, Del Riego et al. reported a sensitivity of 52.6 % for AUS + FNAB and a somewhat lower sensitivity of 46.9 % for AUS alone [20]. Using ultrasound-guided CNB, Solon et al. found a comparable sensitivity of 52.4 % in a series of 210 ALN + women [21]. After having compared FNAB and CNB in a small series of 47 patients, Rao et al. do not advocate the routine use of CNB over FNAB as the sensitivities were comparable (75 % for FNAB and 82 % for CNB), with CNB being considerably more expensive [7]. In another single centre study, Breitbach et al. also found a comparable sensitivity for both biopsy techniques [22]. Other studies, however, report higher sensitivity values for CNB than for FNAB [8,23,24]. In a meta-analysis of 1353 patients in six studies, Balasubramanian et al. conclude that CNB is a superior technique to FNAB for axillary staging, although CNB comes along with a significantly higher complication rate [24]. Their analysis comprised a mix of symptomatic and asymptomatic breast cancers and time trends were not assessed. The sensitivity of FNAB and CNB in our series fall within the range of those reported in the studies mentioned above. As the overall sensitivity of AUS for the detection of ALN+ is often less than 50 %, if clinically relevant, SNB should not be omitted for definitive surgical staging of the axilla in case of normal findings at AUS. SNB may be indicated in women with benign findings at percutaneous axillary biopsy as the latter may yield false negative results [25,26].

Unfortunately, the increased use of CNB at the expense of FNAB did not result in a statistically significantly increased percentage of women with their axillary nodal spread having been confirmed by percutaneous biopsy. Therefore, we do not advocate the routine use of CNB in women with screen-detected breast cancer, also taking into account the significantly higher complication rate of CNB [24].

We found a profound decrease in the proportion of women whose axillary metastases were treated by ALND, from 96.0 % in 2005–2007 to 16.6 % in 2017–2019. A recent meta-analysis reported that ALND patients had a significantly higher prevalence of lymph edema, pain and reduced strength of the upper limb compared to women who had undergone SNB alone [10]. Multicentre, randomised controlled trials show a similar survival for treatment with sentinel lymph node biopsy alone versus treatment with axillary lymph node dissection in patients with minimal or moderate tumour burden in the sentinel nodes [11,12]. Therefore, a routine use of ALND in these patients is not advised anymore, with SNB followed by adjuvant regional lymph node radiation as an alternative to ALND. This radiation increases the risk of breast cancer-related lymph edema to a small extent, with the main risk factor for lymph edema being the type of axillary surgery used [27].

Our study has strengths and limitations. Strengths include the multicentre setting and the large study population with virtually complete follow-up. To our knowledge, this study is the first displaying time-trends in the frequency and yield of axillary biopsy and changes in biopsy procedures of axillary lymph nodes in women with screen-detected breast cancer. On the other hand, our findings on axillary biopsy will be influenced by the fact that we focused on screen-detected cancers, which are usually smaller, less aggressive and less likely to have axillary metastasized disease than symptomatic cancers in screened and non-screened women [28]. Finally, certain tumour characteristics and nodal features may be associated with higher axillary burden, including tumour location, lobular histology and histological grade of the breast cancer, lymphovascular invasion, number of suspicious lymph nodes and increased nodal cortical thickness [17,29,30]. We did not perform a multivariate analysis to examine factors correlated with positive biopsy outcome as the decision of the radiologist to biopsy an axillary lymph node was solely based on the node characteristics at ultrasound.

## 5 Conclusion

In summary, we found that the yield of percutaneous axillary biopsy in case of screen-detected cancer did not improve over the years, despite an increased use of larger biopsy needles. On the other hand, the proportion of ALN + women that underwent ALND significantly dropped, with one out of six ALN + women currently being treated by ALND.

## Ethics approval

Ethical approval was not required for the current study, according to the Dutch Central Committee on Research involving Human Subjects (CCMO).

## Funding

None.

## Author contributions

All authors contributed to the study's conception and design. LD performed material preparation and data collection. LD and AV performed the data-analysis. LD wrote the manuscript's first draft, and all authors commented on subsequent versions. All authors read and approved the final manuscript.

## Data statement

The datasets used and/or analyzed during the current study are available from the corresponding author on reasonable request.

## Declaration of competing interest

None.
